# An epidemiological investigation of porcine circovirus type 2 and porcine circovirus type 3 infections in Tianjin, North China

**DOI:** 10.7717/peerj.9735

**Published:** 2020-08-31

**Authors:** Shuai-Yong Wang, Ying-Feng Sun, Qi Wang, Ling-Xue Yu, Shi-Qiang Zhu, Xiao-Min Liu, Yun Yao, Juan Wang, Tong-Ling Shan, Hao Zheng, Yan-Jun Zhou, Wu Tong, Ning Kong, Guang-Zhi Tong, Hai Yu

**Affiliations:** 1 Shanghai Veterinary Research Institute, Chinese Academy of Agricultural Sciences, Shanghai, China; 2 College of Animal Science and Veterinary Medicine, Tianjin Agricultural University, Tianjin, China; 3 Jiangsu Co-innovation Center for Prevention and Control of Important Animal Infectious Diseases and Zoonoses, Yangzhou, China

**Keywords:** Porcine circovirus type 3, Double PCR, Coinfection, Complete genome sequences

## Abstract

Novel porcine circovirus type 3 (PCV3), first identified in the United States, has been detected in many other countries. Porcine circovirus is associated with postweaning multisystemic wasting syndrome, reproductive failure, congenital tremors, and other clinical symptoms. In this study, we established a double polymerase chain reaction assay for detecting both porcine circovirus type 2 (PCV2) and PCV3. This is the first study to detect and characterize the PCV3 genome in the Tianjin region of North China. We collected a total of 169 tissue samples from seven farms between 2016 and 2018. The PCV3-positive rate of all tissue samples was 37.3% (63/169) and the rate of PCV2 and PCV3 coinfection was 14.8% (25/169). PCV2 and PCV3 coinfections with more serious clinical symptoms were found in only three farms. We sequenced three PCV3 strains selected from tissue samples that were positively identified. The complete genome sequences of the three strains shared 97.6–99.4% nucleotide identities with the PCV3 strains in GenBank. Our results showed the extent of PCV3’s spread in Tianjin, and the need to further study PCV3’s pathobiology, epidemiology, isolation, and coinfection.

## Introduction

Porcine circovirus (PCV), a member of the genus *Circovirus*, is a single-stranded DNA virus that can autonomously replicate. PCV has a genome size of 1,700 bp and two basic open reading frames (ORFs) in its DNA sequence called rep and cap. The rep and cap ORFs mainly code for the replicase and capsid proteins, respectively (Hamel, Lin & Nayar, 1998; Harms et al., 2001; Meng, 2013). Porcine circovirus type 1 (PCV1) was first found in 1974 as a nonpathogenic contaminant in PK-15 cells (Tischer et al., 1986). Porcine circovirus type 2 (PCV2) is a variant of PCV1 first found in piglets in 1998 (Allan et al., 1998; Tischer et al., 1995). Previous studies have shown that the nucleotide sequence homology between PCV2 and PCV1 is only about 68%, and that the two viruses have different antigens and phenotypes (Allan et al., 1994; Hamel, Lin & Nayar, 1998; Meehan et al., 1998). PCV2 is associated with many diseases including reproductive disorders, enteric diseases, respiratory diseases, and postweaning multisystemic wasting syndrome (PMWS). These diseases are known as porcine circovirus associated diseases (PCVAD) in the United States, and porcine circovirus diseases (PCVD) in Europe (Harms et al., 2001; Krakowka et al., 2000; Opriessnig, Meng & Halbur, 2007; Segales, 2012).

In 2016, novel porcine circovirus type 3 (PCV3) was first reported in the United States. Palinski et al. (2017) observed that PCV3’s putative cap ORF encoded a 214-aa protein that was 36–37% identical to PCV2. PCV3 is associated with cardiac, multisystemic (Phan et al., 2016), and reproductive failure, as well as porcine dermatitis and nephropathy syndrome (Palinski et al., 2017). PCV3’s complete sequence size is 2,000 bp with three major ORFs (Palinski et al., 2017; Phan et al., 2016). PCV3 has been detected in pigs across many countries (Faccini et al., 2017; Hayashi et al., 2018; Klaumann et al., 2019; Stadejek et al., 2017; Sukmak et al., 2019; Ye et al., 2018). Although there have been some reports of PCV3 in South China, no cases of PCV3 in Tianjin, North China have been reported (Chen et al., 2017; Fan et al., 2017; Ku et al., 2017; Liu et al., 2019; Qi et al., 2019; Shen et al., 2018; Wen et al., 2018; Xu et al., 2018; Zheng et al., 2017; Zou et al., 2018).

This is the first investigation of the spread of PCV3 in Tianjin, North China. We looked at seven farms in seven of its regions (Jizhou, Baodi, Wuqing, Ninghe, Dongli, Jinghai, and Binhai) between 2016 and 2018. Since pig breeding and farming are major industries in Tianjin, it is crucial to study the epidemic nature of PCV3 in North China.

## Materials and Methods

### Sample collection and clinical symptoms

We collected a total of 169 pig tissue samples (including hearts, livers, spleens, lungs, and kidneys) from seven farms (in Jizhou, Baodi, Wuqing, Ninghe, Dongli, Jinghai, and Binhai) in Tianjin, North China between December 2016 and May 2018. All samples were stored at −80 °C. We selected 10 pigs from each farm for a total of 70 pigs. The selected pig’s major clinical symptoms included fever, cough, anorexia, depression, an increase in mortality rate, and a decrease in conception rate. We compared the clinical symptoms from each farm to determine the distinction between mild and severe cases. In this study, Animal committee of Shanghai Veterinary Research Institute provided full approval for this research (permit number SHVRI-Pig-20161206-05), field experiments were approved by the Scientific Research Office of Shanghai Veterinary Research Institute (permit number SHVRI-Pig-160417-19).

### Polymerase chain reaction assay

The collected tissue samples were cut into small pieces (0.1 cm × 0.1 cm) and packed into two mL Eppendorf tubes. After three freeze-thaw cycles, the small pieces of tissue were ground thoroughly with one mL of Dulbecco’s modified Eagle’s medium (DMEM; Hyclone, Beijing, China) and centrifuged at 3,000×*g* for 10 min at 4 °C. The supernatants were stored at −80 °C. We extracted nucleic acid using a DNeasy® Blood & Tissue Kit (Qiagen, Hilden, Germany) according to the manufacturer’s instructions.

A double PCR assay was established to detect PCV2 and PCV3. Using bioinformatics analysis, we designed and synthesized two pairs of specific primers to detect PCV2 and PCV3 (Table 1) from a commercial source (GENEWIZ). Amplicon products were cloned into a PLB vector (Tiangen, Beijing, China) and sequenced by Sangon Biotech Company (Shanghai, China). The complete PCV2 and PCV3 sequences were amplified using the primer pairs PCV2-F (5″-ATCCACGGAGGAAGGGGGCCAGTT-3″) and PCV2-R (5″-GTGGATTGTTCTGTAGCATTCTTCCA-3″) (Guo et al., 2010), PCV3-1F (5″-CACCGTGTGAGTGGATATAC-3″) and PCV3-1R (5″-CAAACCCACCCTTAACAG-3″), PCV3-2F (5″-GTCGTCTTGGAGCCAAGTG-3″) and PCV3-2R (5″-CGACCAAATCCGGGTAAGC-3″), PCV3-3F (5″-TGTTGTACCGGAGGAGTG-3″) and PCV3-3R (5″-TGCCGGGTAATACTAGCC-3″), and PCV3-4F (5″-GAAGTTGCGGAGAAGATG-3″) and PCV3-4R (5″-TCCAAGACGACCCTTATG-3″) (Palinski et al., 2017). The amplicon products were also cloned into a PLB vector (Tiangen, Beijing, China) and sequenced by Sangon Biotech Company (Shanghai, China).

**Table 1:** Primers for PCV2 and PCV3 specific target gene amplification by the double PCR.

Virus	Primer sequence (5′ → 3′)	Product length (bp)	Annealing temperature (°C)
PCV2	F: ATAGGGGTCATAGGTTAGGGCATT	305	56
	R: GAAAAATGGCATCTTCAACACCCG		
PCV3	F: GGAGGTTCACTAAGGTTGTTTGTT	767	58
	R: ACCACTTCATTACCCGCCTAAACGA		

We carried out a single PCR for PCV2 and PCV3 in a 50 μl mixture containing 25 μl Takara La Taq™ DNA polymerase (5 U/μl), one μl forward primer (10 mmol/μl), one μl reverse primer (10 mmol/μl), one μl DNA template, and 22 μl distilled water. The DEPC-treated water was used as a template for negative control reactions. The reaction was performed under the following conditions: initial denaturation at 94 °C for 3 min; 30 cycles of 94 °C for 30 s, 55 °C (PCV2) or 58 °C (PCV3) for 30 s, and 72 °C for 1 min; and the final extension at 72 °C for 10 min. We detected PCR products by electrophoresing through 1% agarose gel in 1× TAE buffer. Each specific viral target fragment was cloned into the plasmid PLB vector (Tiangen, Beijing, China) and sequenced (Invitrogen, Carlsbad, CA, USA).

We optimized the double PCR reaction by changing a single condition and leaving the other conditions unchanged. We also experimentally optimized the Takara La Taq™ DNA polymerase from 1 to 5 U, each primer from 2 to 20 pmol, the annealing temperature from 50 to 70 °C, and the number of cycles from 25 to 40. We detected PCR products by electrophoresing through 1% agarose gel in 1× TAE buffer. To ensure the sensitivity of the double PCR assay and to determine the limits of detection, we diluted the recombinant plasmid of the complete DNA sequences of the two viruses using a 10-fold series. The following formula was used to calculate the number of gene copies per μl in each dilution: copies/μl = (6.02 × 10^23^) × (Plasmid concentration (ng/μl) × 10^−9^)/(DNA length (bp) × 660) (Yue et al., 2009). To confirm the specificity of the double PCR assay, we also tested for pseudorabies virus (PRV), porcine reproductive and respiratory syndrome (PRRSV), classical swine fever virus (CSFV), Japanese encephalitis virus (JEV), porcine epidemic diarrhea virus (PEDV), swine influenza virus (SIV), porcine parvovirus (PPV), and transmissible gastroenteritis virus (TGEV) using the primers listed earlier in Table 1.

### Detecting viruses in clinical samples using PCR assays

We extracted the DNA from the 169 collected samples using the methods described earlier. The samples were tested for PCV2 and PCV3 using single PCR and double PCR. Additionally, we prepared the extracted nucleic acid of the collected samples and used PCR to detect other common porcine viruses PCV1, PRV, PRRSV, CSFV, JEV, PEDV, SIV, PPV, and TGEV. The primers for PRV, PRRSV, CSFV, JEV and PPV were referred to Zeng et al. (2014), the primers for PEDV and TGEV were referred to Ogawa et al. (2009), the primers for SIV were referred to Poddar (2002) and the primers for PCV1 were referred to Yu et al. (2018). All the primers were synthesized from a commercial source (GENEWIZ, South Plainfield, NJ, USA).

### Virus isolation

Virus isolation was carried out in a PK-15 cell lines were obtained from the Cell Bank of the Chinese Academy of Sciences (Shanghai, China). The cells were resuspended in 15 ml of DMEM (Hyclone, Beijing, China) containing 10% fetal bovine serum (FBS) (Hyclone, Beijing, China) and inoculated with 500 μl filtered tissue sample supernatants. These cultures were then incubated at 37 °C for 18 h in a 10% CO_2_ atmosphere, and the resulting semiconfluent monolayers were treated with 300 mM D-(+)-glucosamine hydrochloride (Sigma, St. Louis, MO, USA) and incubated for an additional 48–72 h at 37 °C (Allan et al., 1998). All PCV2- and PCV3-positive samples were used to isolate the viruses. After three passages, we used the freeze-thawed third generation PK-15 cell culture to extract DNA and the DNA was detected by using the established double PCR assay.

### Phylogenetic analysis

Phylogenetic trees were reconstructed using the maximum-likelihood method with a MEGA 6.0 software bootstrap analysis of 1,000 replicates.

## Results

### Optimizing double PCR

**Figure 1:**
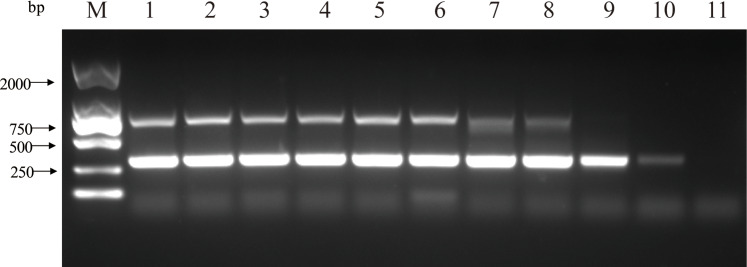
Influence of a range of annealing temperatures in the efficiency of amplification for two close related viruses by double PCR. M: DL2000 DNA Marker; 1: 50 °C; 2: 52 °C; 3: 54 °C; 4: 56.4 °C; 5: 58.8 °C; 6: 61.2 °C; 7: 63.6 °C; 8: 66 °C; 9: 68 °C; 10: 70 °C; 11: negative control.

**Figure 2:**
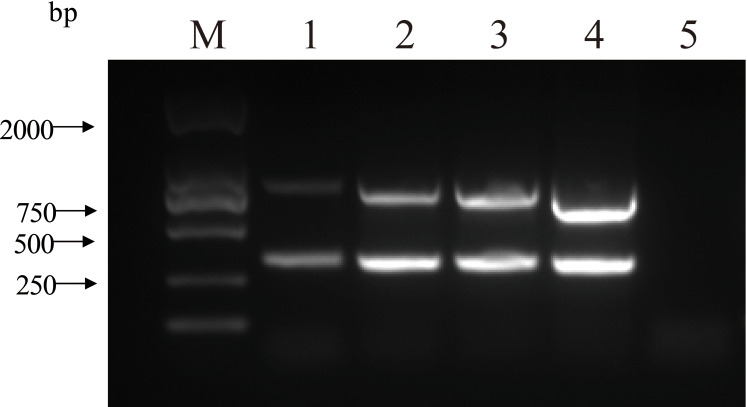
Influence of a range of number of cycles in the efficiency of amplification for two close related viruses by double PCR. M: DL2000 DNA Marker; 1: 40 cycles; 2: 35 cycles; 3: 30 cycles; 4: 25 cycles; 5: negative control.

The double PCR using two pairs of primers for PCV2 and PCV3 produced specific amplicon lengths of 305 bp and 767 bp, respectively. The optimal annealing temperature was 58.8 °C (Fig. 1) and the optimal number of cycles was 30 (Fig. 2). Other optimal double PCR conditions were 4.5 U of La Taq™ DNA polymerase and 10 pmol of primer. Under optimized conditions, the double PCR effectively amplified the two viruses.

### Sensitivity and specificity of single and double PCR

**Figure 3:**
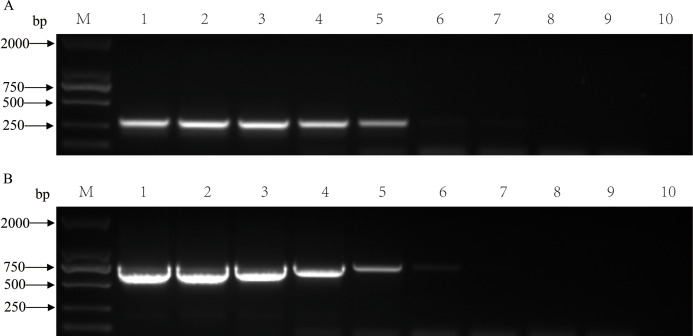
Sensitivity of single PCR for detecting single viral nucleic acid of PCV2 or PCV3 by diluting the nucleic acid using a 10-fold series. (A) Single PCR sensitivity for PCV2. M: DL2000 DNA Marker; 1: 10^9^ copies; 2: 10^8^ copies; 3: 10^7^ copies; 4: 10^6^ copies; 5: 10^5^ copies; 6: 10^4^ copies; 7: 10^3^ copies; 8: 10^2^ copies; 9: 10^1^ copies; 10: negative control. (B) Single PCR sensitivity for PCV3. M: DL2000 DNA Marker; 1: 10^9^ copies; 2: 10^8^ copies; 3: 10^7^ copies; 4: 10^6^ copies; 5: 10^5^ copies; 6: 10^4^ copies; 7: 10^3^ copies; 8: 10^2^ copies; 9: 10^1^ copies; 10: negative control.

**Figure 4:**
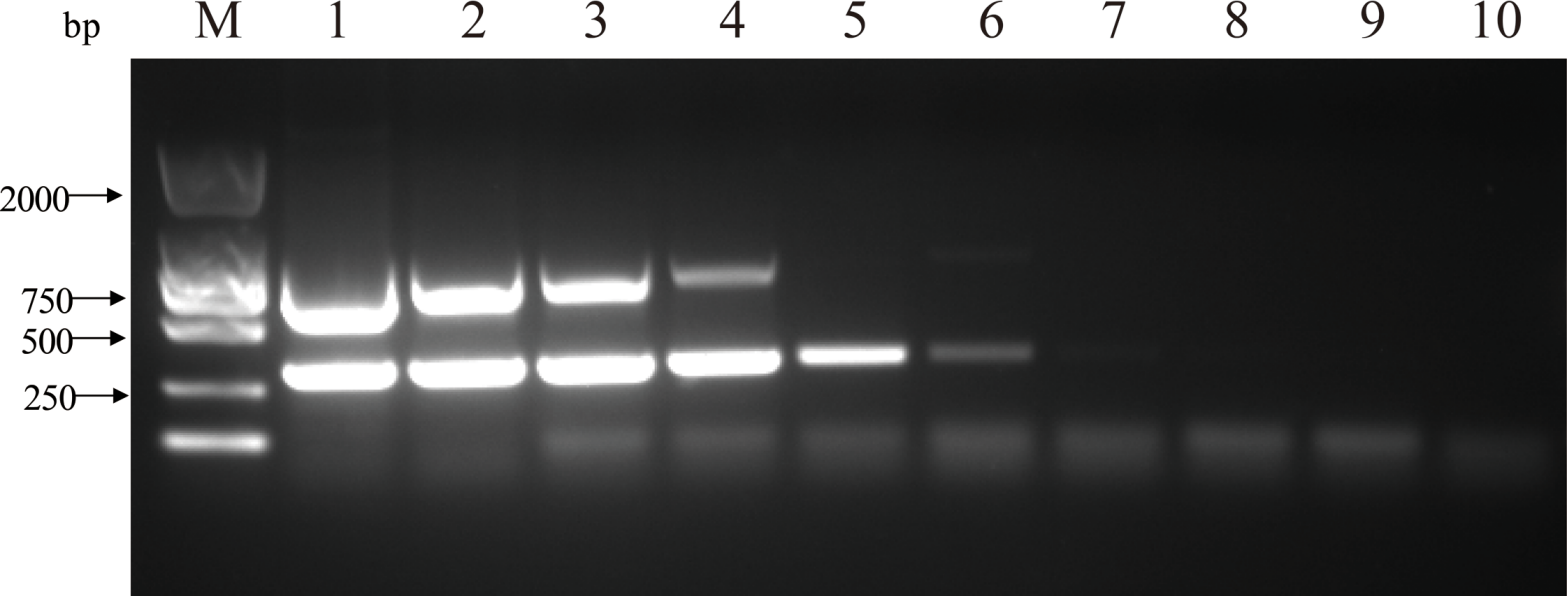
Sensitivity of double PCR for detecting PCV2 and PCV3 by diluting the nucleic acid using a 10-fold series. M: DL2000 DNA Marker; 1: 10^9^ copies; 2: 10^8^ copies; 3: 10^7^ copies; 4: 10^6^ copies; 5: 10^5^ copies; 6: 10^4^ copies; 7: 10^3^ copies; 8: 10^2^ copies; 9: 10^1^ copies; 10: negative control.

**Figure 5:**
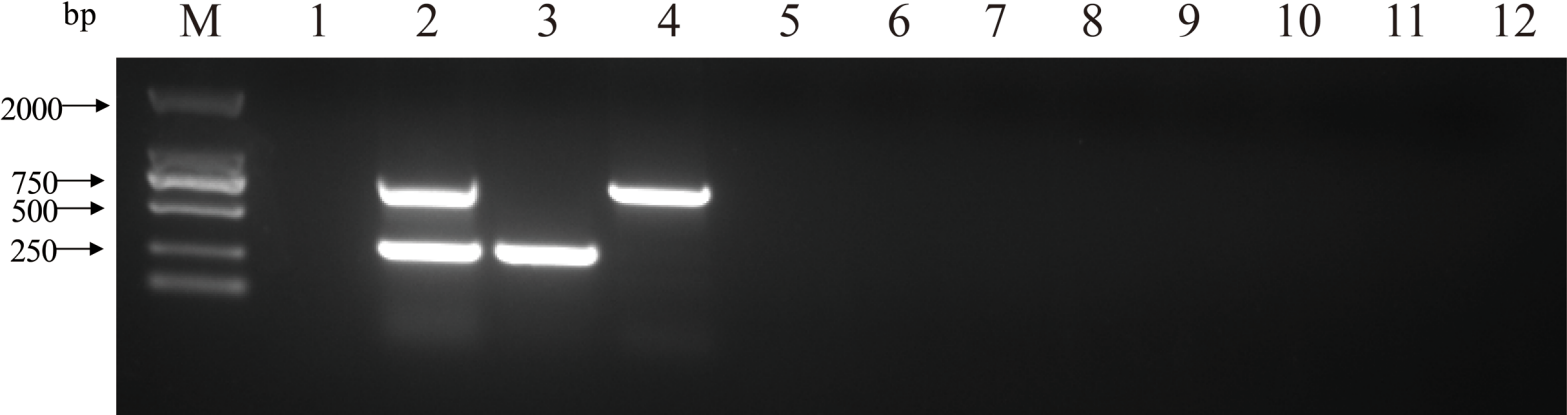
Specificity of double PCR for detecting PCV2, PCV3, PRV, PRRSV, CSFV, JEV, PEDV, SIV, PPV and TGEV. M: DL2000 DNA Marker; 1: negative control; 2: PCV2 and PCV3; 3: PCV2; 4: PCV3; 5: PRV; 6: PRRSV; 7: CSFV; 8: JEV; 9: PEDV; 10: SIV; 11: PPV; 12: TGEV.

The nucleic acids of the two viruses were diluted 10-fold and mixed to form templates for single and double PCR sensitivity assays. The single PCR detection limit copy numbers were 1.44 × 10^5^ and 2.23 × 10^5^ for PCV2 and PCV3, respectively (Fig. 3). The detection limits of the double PCR were 1.44 × 10^4^ and 2.23 × 10^6^ copies for PCV2 and PCV3, respectively (Fig. 4). We evaluated the specificity of the two pairs of primers using double PCR. As shown in Fig. 5, the double PCR was specific to the target viral agent. No amplicons were produced with the other agents (PRV, PRRSV, CSFV, JEV, PEDV, SIV, PPV or TGEV) or with ddH2O. All mentioned amplicons and plasmids were sequenced to control for potential false positive results. The double PCR amplicon sequences corresponded to 305 bp for PCV2 and 767 bp for PCV3.

### Detecting clinical samples using PCR assays and clinical symptom records

In this study, we analyzed a total of 169 collected samples for PCV2 and PCV3. Sixty-three tissue samples were positive for PCV3, with a 37.3% positive rate. Forty-eight tissue samples were positive for PCV2, with a 28.4% positive rate. Additionally, 25 tissue samples were coinfected with PCV2 and PCV3, and the coinfection rate was 14.8%. We found no other common porcine viruses among the 169 tissue samples. The specific clinical symptoms were different across the seven farms (Table 2) and coinfection was only found in Farm-D, Farm-E, and Farm-G.

**Table 2:** List of clinical symptoms at the seven farms in Tianjin.

Farm name	Fever	Cough	Anorexic	Depression	Increase in mortality rate	Decrease in conception rate
Farm-A^a^	+	–	+	–	+	+
Farm-B^b^	+	–	+	–	+	–
Farm-C^a^	–	–	–	+	–	+
Farm-D^c^	+	–	+	++	++	+
Farm-E^c^	++	+	+	++	+	++
Farm-F^a^	+	–	+	–	–	+
Farm-G^c^	++	++	+	++	++	++

**Notes:**

a Only PCV3 infection was detected on the farm.

b Only PCV2 infection was detected on the farm.

c Both PCV2 and PCV3 were detected on the farm.

+/−, positive or negative appearance of clinical symptoms; ++, degree of severity of the demonstrated clinical symptoms.

### Virus isolation

After three passages through the PK-15 cell culture, the cell culture DNA was extracted and detected using single and double PCR. We isolated six strains of the virus, all of which were PCV2 strains.

### Phylogenetic analysis

**Figure 6:**
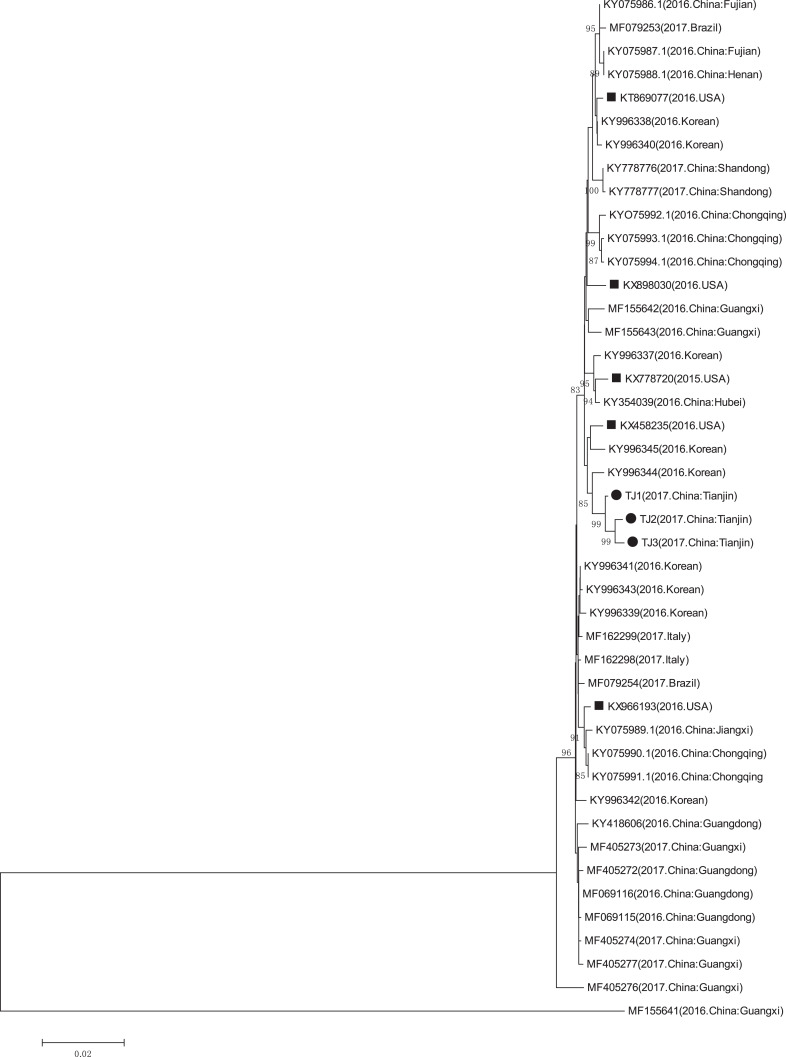
Phylogenetic tree based on the complete genome sequences of PCV3 obtained from GenBank database and three sequences detected in this study. The Phylogenetic tree was constructed using the *p*-distance-based neighbor-joining method in MEGA 6.0 software. Bootstrap values were calculated with 1,000 replicates. Black solid circles indicate the strains from Tianjin. Black solid squares indicate the strains from the United States. Scale bars indicate nucleotide substitutions per site.

**Figure 7:**
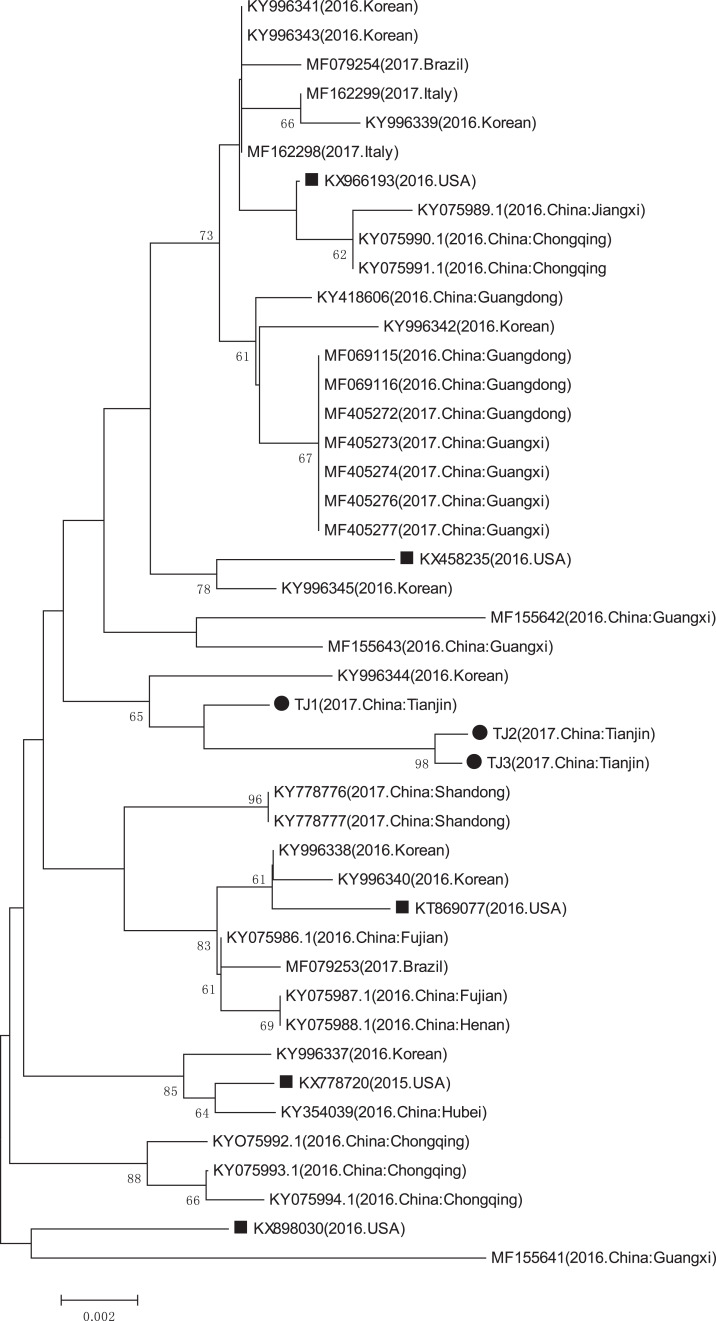
Phylogenetic tree based on the cap gene sequences of PCV3 obtained from GenBank database and three sequences detected in this study. The phylogenetic tree was based on the partial capsid gene sequences. The phylogenetic tree was constructed using the *p*-distance-based neighbor-joining method in MEGA 6.0 software. Bootstrap values were calculated with 1,000 replicates. Black solid circles indicate the strains from Tianjin. Black solid squares indicate the strains from the United States. Scale bars indicate nucleotide substitutions per site.

The GenBank accession numbers of the three PCV3 strains identified in this study are MN790774, MN790775, and MN790776. The genomic homology of the complete genome and the cap gene of the three identified PCV3 strains with that of the other PCV3 strains (except the PCV3-China/GX2016-1 strain which had a deletion (Wen et al., 2018)), were in the 97.6–99.4% and 97.1–99.1% ranges, respectively. The complete genome and cap gene shared 99.5–99.6% and 99.1–99.8% homology between the three identified PCV3 strains, respectively. The phylogenetic analyses of the complete genome and the cap gene showed that the three PCV3 strains from Tianjin were closely related to the PCV3/KU-1608 (KY996344) strain first reported in South Korea (Figs. 6 and 7) (Kwon et al., 2017) and the genomic homology of the complete genome and the cap gene of the three identified PCV3 strains with PCV3/KU-1608 (KY996344) were up to 99.4% and 99.1%. Additionally, the cap gene length of all three PCV3 strains measured 645 bp.

## Discussion

PCV3 cases have been reported in many countries. This new virus is associated with reproductive failure, cardiac, multisystemic inflammation, congenital tremors, and other porcine diseases (Chen et al., 2017; Palinski et al., 2017; Phan et al., 2016; Wen et al., 2018). PCVAD presents an immense economic burden to the global swine industry (Meng, 2013), creating a high demand for rapid and precise diagnostic methods. Additionally, PCV1 and PCV2 diagnostic methods have been established and are widely used (Li et al., 2013). In this study, we established a double PCR assay to detect PCV2 and PCV3 in individual reaction systems. The double PCR assay can increase the efficiency of sample detection, help monitor incident rates, and confirm the prevalence of the two viruses. The rapid development of the pig breeding industry calls for a better understanding of PCV3 prevalence. We collected a total of 169 tissue samples from Tianjin. The PCV3 positive rate was 37.3%, similar to the national average in China (Chen et al., 2017; Ku et al., 2017; Wen et al., 2018; Zheng et al., 2017). The phylogenetic tree showed that the three PCV3 strains were all in the same clade as another PCV3 strain first found in Korea (Kwon et al., 2017) and the genomic homology of the complete genome and the cap gene of the three identified PCV3 strains with PCV3/KU-1608 (KY996344) were up to 99.4% and 99.1%, perhaps due to geographic distribution.

Previous studies have shown that viral coinfection can cause more damage and/or viral replication in animals (Ellis et al., 2000; Meng, 2013; Opriessnig & Halbur, 2012; Pogranichniy et al., 2002; Ramamoorthy & Meng, 2009). We found similar results in the seven farms in this study. The clinical symptoms of Farm-D, Farm-E, and Farm-G were more severe than the other four farms, and PCV2 and PCV3 coinfection was only found on these three farms. It would be interesting to determine whether coinfection can cause more severe clinical symptoms than single PCV2 or PCV3 infection.

However, our isolation of the PCV3 virus failed. The suspension isolated from PCV3-positive tissue samples was inoculated in PK-15 cells and went through three continuous passages (Tischer et al., 1995). The results of the serial passage tests showed that PCV3 could not be isolated by this method. We conjectured that the PK-15 cell line is not a suitable host for PCV3. To find a new host cell line for PCV3 are need to further research.

## Conclusions

We investigated the presence of PCV3 and the rate of PCV2 and PCV3 coinfection on farms in Tianjin, North China using an established double PCR assay. The results indicated the prevalence of PCV3 and PCV2 in swine herds. To better understand the impact of this agriculturally important pathogen, future studies should explore the pathobiology and epidemiology of PCV3.

## Supplemental Information

10.7717/peerj.9735/supp-1 Supplemental Information 1 DNA sequence of MN790774 (PCV3-TJ1).

10.7717/peerj.9735/supp-2 Supplemental Information 2 DNA sequence of MN790775 (PCV3-TJ2).

10.7717/peerj.9735/supp-3 Supplemental Information 3 DNA sequence of MN790776 (PCV3-TJ3).

10.7717/peerj.9735/supp-4 Supplemental Information 4 Effect of annealing temperature on detection of two viruses by double PCR. Raw data of full-length image of gel

10.7717/peerj.9735/supp-5 Supplemental Information 5 Effect of number of cycles of double PCR. Raw data of full-length image of gel

10.7717/peerj.9735/supp-6 Supplemental Information 6 Sensitivity of single PCR for PCV2. Raw data of full-length image of gel

10.7717/peerj.9735/supp-7 Supplemental Information 7 Sensitivity of single PCR for PCV3. Raw data of full-length image of gel

10.7717/peerj.9735/supp-8 Supplemental Information 8 Sensitivity of double PCR for each virus. Raw data of full-length image of gel

10.7717/peerj.9735/supp-9 Supplemental Information 9 Specificity of double PCR for the differentiation. Raw data of full-length image of gel

10.7717/peerj.9735/supp-10 Supplemental Information 10 PCV3 sequences obtained from GenBank that were used in this study.

## Additional Information and Declarations

### Field Study Permissions

The following information was supplied relating to field study approvals (i.e., approving body and any reference numbers): Field experiments were approved by the Scientific Research Office of Shanghai Veterinary Research Institute (SHVRI-Pig-160417-19).

### Data Availability

The following information was supplied regarding data availability: The raw measurements are available in the Supplemental Files. The DNA sequences are available as Supplemental Files and available at GenBank: MN790774–MN790776.
