# Supplementary figures and images for: An epidemiological investigation of porcine circovirus type 2 and porcine circovirus type 3 infections in Tianjin, North China
Source: PeerJ. 2020 Aug 31;8:e9735. doi: 10.7717/peerj.9735 (PMC7469938; doi:10.7717/peerj.9735)

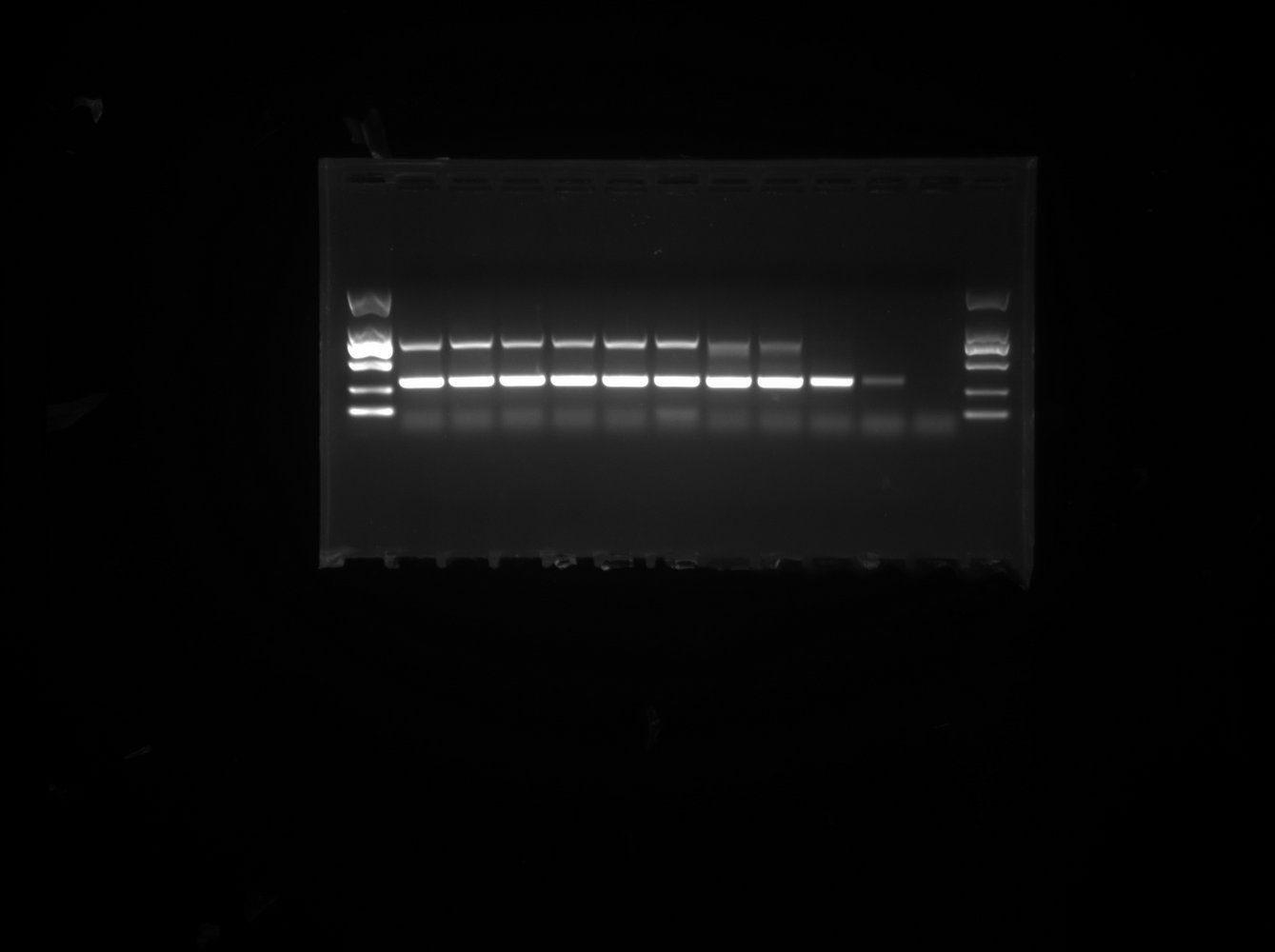

Supplement: Supplemental Information 4 — Raw data of full-length image of gel [file peerj-08-9735-s004.jpg]

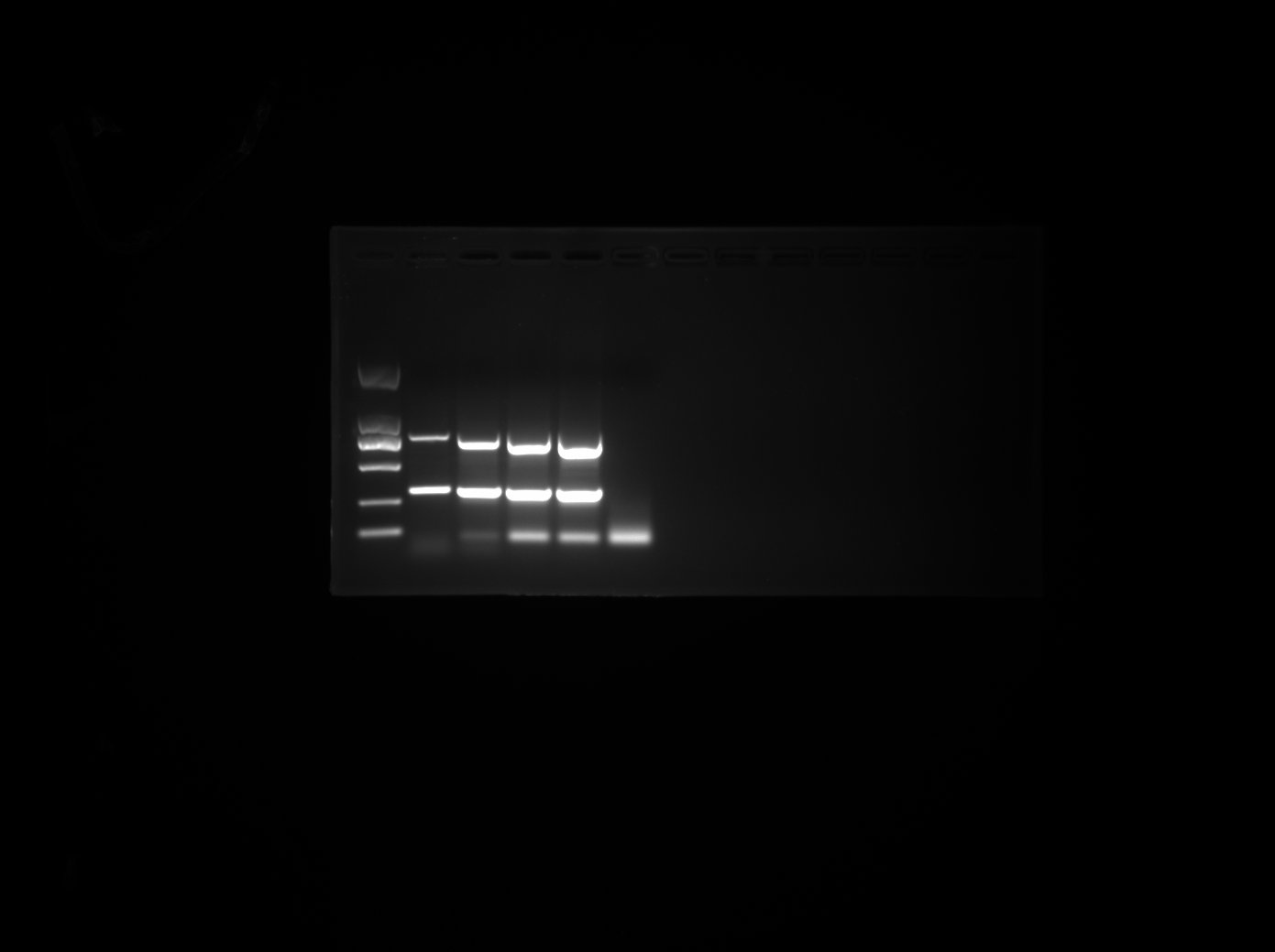

Supplement: Supplemental Information 5 — Raw data of full-length image of gel [file peerj-08-9735-s005.jpg]

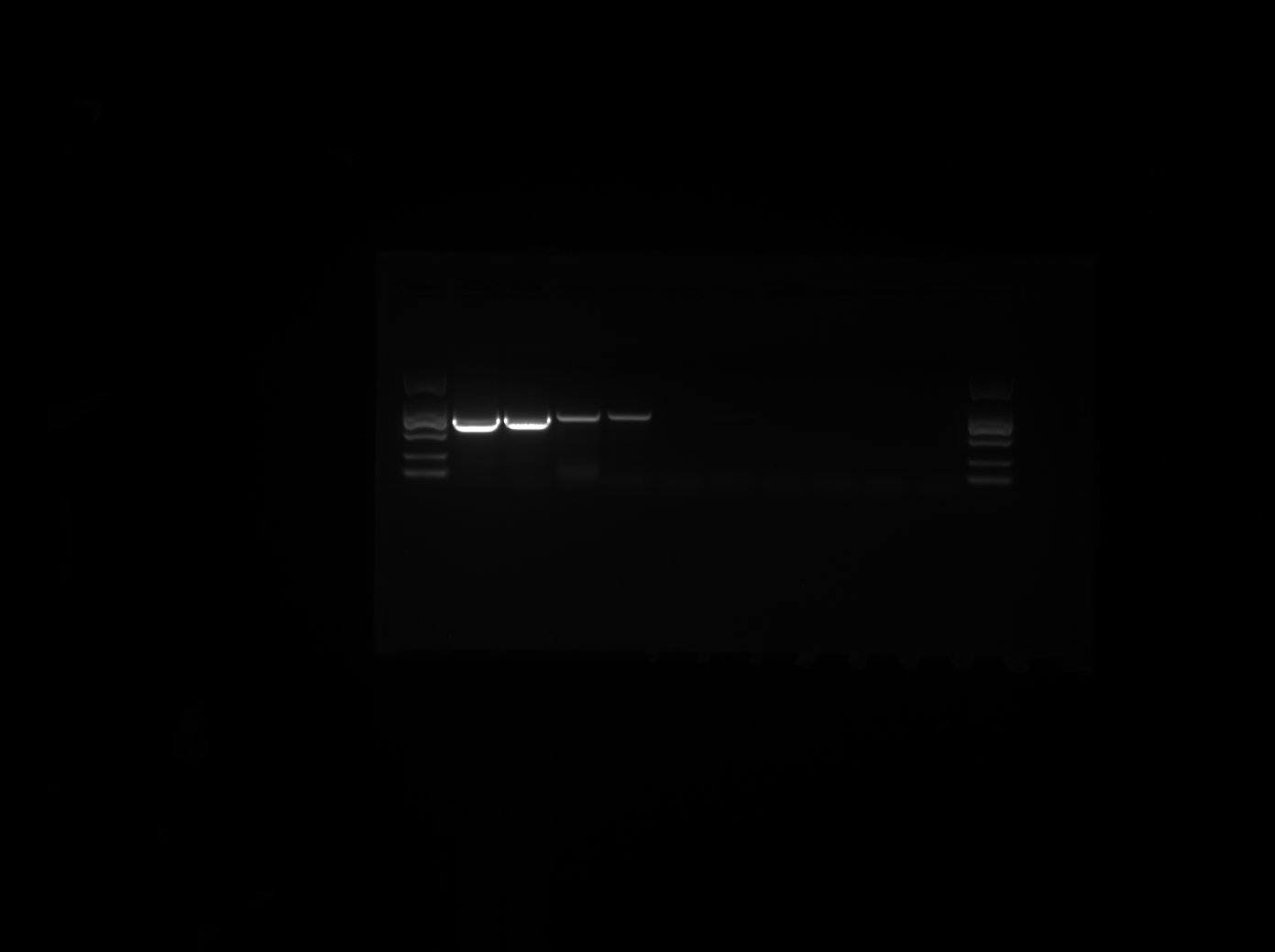

Supplement: Supplemental Information 6 — Raw data of full-length image of gel [file peerj-08-9735-s006.jpg]

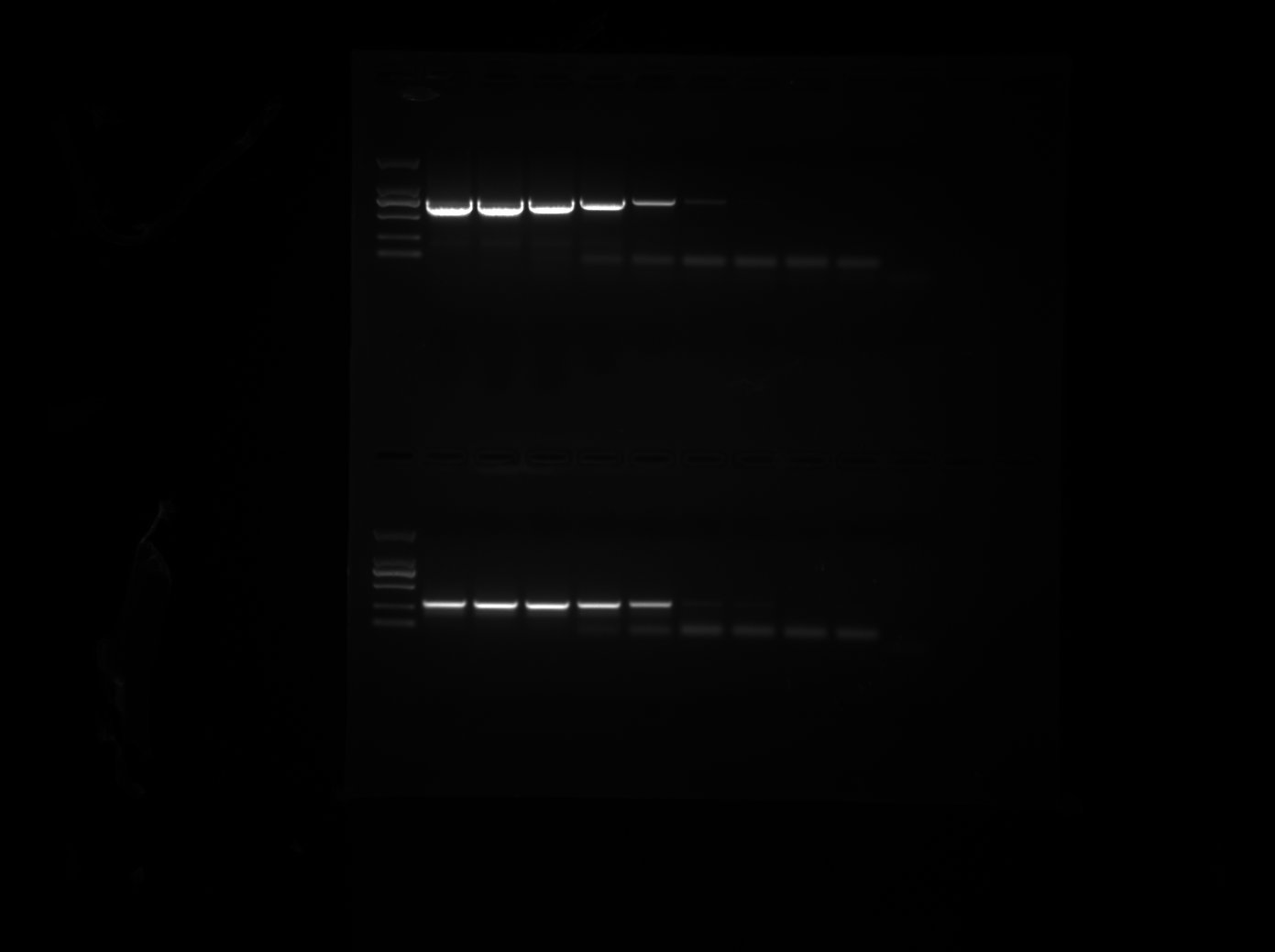

Supplement: Supplemental Information 7 — Raw data of full-length image of gel [file peerj-08-9735-s007.jpg]

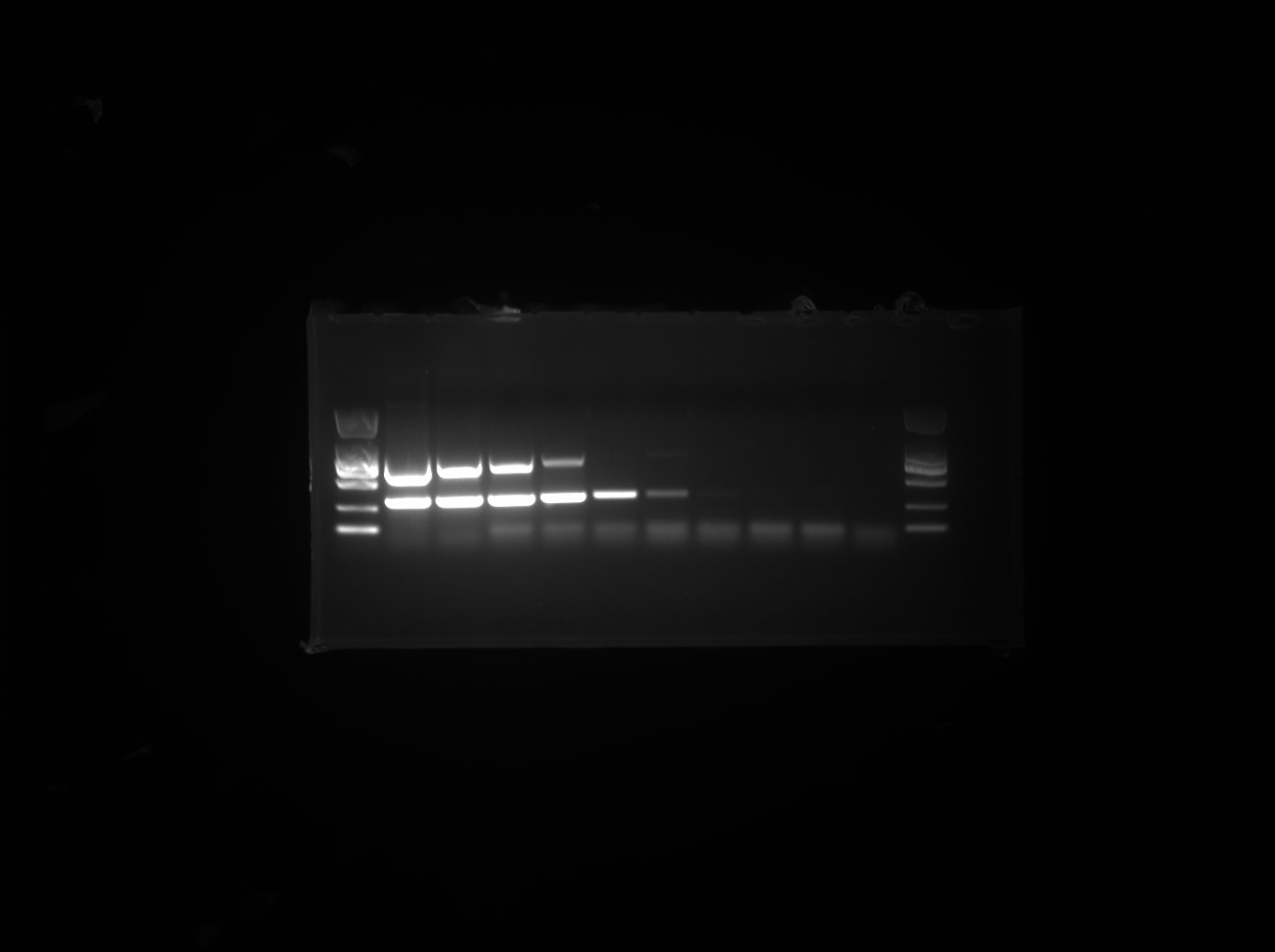

Supplement: Supplemental Information 8 — Raw data of full-length image of gel [file peerj-08-9735-s008.jpg]

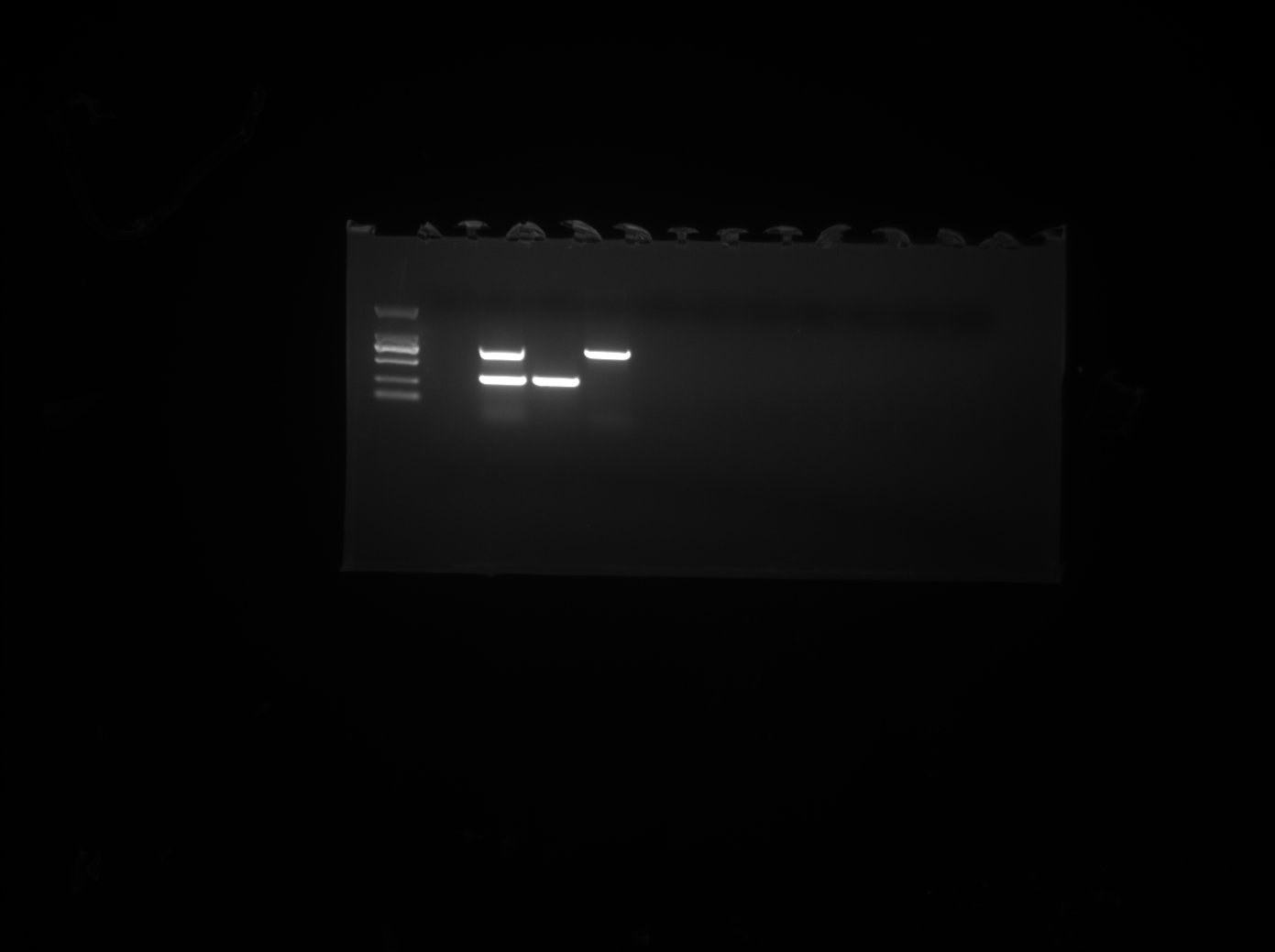

Supplement: Supplemental Information 9 — Raw data of full-length image of gel [file peerj-08-9735-s009.jpg]
