# Supplementary material for: An epidemiological investigation of porcine circovirus type 2 and porcine circovirus type 3 infections in Tianjin, North China
Source: PeerJ. 2020 Aug 31;8:e9735. doi: 10.7717/peerj.9735 (PMC7469938; doi:10.7717/peerj.9735)
Supplement: Supplemental Information 10 [file peerj-08-9735-s010.docx]

**TABLE 2**

| GenBank accession number | Years | Country origin | Genotype | References | Name |
| --- | --- | --- | --- | --- | --- |
| KT869077 | 2016 | USA | PCV3 | Palinski et al. (2017) | 29160 |
| KX458235 | 2016 | USA | PCV3 | Palinski et al. (2017) | 2164 |
| KX778720 | 2015 | USA | PCV3 | Phan et al. (2016) | PCV3-US/MO2015 |
| KX898030 | 2016 | USA | PCV3 | Phan et al. (2016) | PCV3-US/MN2016 |
| KX966193 | 2016 | USA | PCV3 | Phan et al. (2016) | PCV3-US/SD2016 |
| KY354039 | 2016 | China | PCV3 | Fan et al. (2017) | PCV3/CN/Hubei-618/2016 |
| KY418606 | 2016 | China | PCV3 | Shen et al. (2017) | PCV3-China/GD2016 |
| KY778776 | 2017 | China:Shandong | PCV3 | Zheng et al. (2017) | PCV3/CN/Shandong-1/201703 |
| KY778777 | 2017 | China:Shandong | PCV3 | Zheng et al. (2017) | PCV3/CN/Shandong-2/201703 |
| MF069115 | 2016 | China:Guangdong | PCV3 | Chen et al. (2017) | PCV3/CN/GDLC1/2016 |
| MF069116 | 2016 | China:Guangdong | PCV3 | Chen et al. (2017) | PCV3/CN/GDHE2/2016 |
| MF405272 | 2017 | China:Guangdong | PCV3 | Chen et al. (2017) | PCV3/CN/GDBL1/2017 |
| MF405273 | 2017 | China:Guangxi | PCV3 | Chen et al. (2017) | PCV3/CN/GXHJ1/2017 |
| MF405274 | 2017 | China:Guangxi | PCV3 | Chen et al. (2017) | PCV3/CN/GXLJ2/2017 |
| MF405276 | 2017 | China:Guangxi | PCV3 | Chen et al. (2017) | PCV3/CN/GXLJ1/2017 |
| MF405277 | 2017 | China:Guangxi | PCV3 | Chen et al. (2017) | PCV3/CN/GXHJ2/2017 |
| KY075986.1 | 2016 | China:Fujian | PCV3 | Ku et al. (2017) | PCV3/CN/Fujian-5/2016 |
| KY075987.1 | 2016 | China:Fujian | PCV3 | Ku et al. (2017) | PCV3/CN/Fujian-12/2016 |
| KY075988.1 | 2016 | China:Henan | PCV3 | Ku et al. (2017) | PCV3/CN/Henan-13/2016 |
| KY075989.1 | 2016 | China:Jiangxi | PCV3 | Ku et al. (2017) | PCV3/CN/Jiangxi-62/2016 |
| KY075990.1 | 2016 | China:Chongqing | PCV3 | Ku et al. (2017) | PCV3/CN/Chongqing-147/2016 |
| KY075991.1 | 2016 | China:Chongqing | PCV3 | Ku et al. (2017) | PCV3/CN/Chongqing-148/2016 |
| KY075992.1 | 2016 | China:Chongqing | PCV3 | Ku et al. (2017) | PCV3/CN/Chongqing-150/2016 |
| KY075993.1 | 2016 | China:Chongqing | PCV3 | Ku et al. (2017) | PCV3/CN/Chongqing-155/2016 |
| KY075994.1 | 2016 | China:Chongqing | PCV3 | Ku et al. (2017) | PCV3/CN/Chongqing-156/2016 |
| MF162298 | 2017 | Italy | PCV3 | Faccini et al. (2017) | PCV3-IT/CO2017 |
| MF162299 | 2017 | Italy | PCV3 | Faccini et al. (2017) | PCV3-IT/MN2017 |
| MF079253 | 2017 | Brazil | PCV3 | Tochetto et al. (2017) | PCV3-BR/RS/6 |
| MF079254 | 2017 | Brazil | PCV3 | Tochetto et al. (2017) | PCV3-BR/RS/8 |
| KY996337 | 2016 | Korean | PCV3 | Kwon et al. (2017) | PCV3/KU-1601 |
| KY996338 | 2016 | Korean | PCV3 | Kwon et al. (2017) | PCV3/KU-1602 |
| KY996339 | 2016 | Korean | PCV3 | Kwon et al. (2017) | PCV3/KU-1603 |
| KY996340 | 2016 | Korean | PCV3 | Kwon et al. (2017) | PCV3/KU-1604 |
| KY996341 | 2016 | Korean | PCV3 | Kwon et al. (2017) | PCV3/KU-1605 |
| KY996342 | 2016 | Korean | PCV3 | Kwon et al. (2017) | PCV3/KU-1606 |
| KY996343 | 2016 | Korean | PCV3 | Kwon et al. (2017) | PCV3/KU-1607 |
| KY996344 | 2016 | Korean | PCV3 | Kwon et al. (2017) | PCV3/KU-1608 |
| KY996345 | 2016 | Korean | PCV3 | Kwon et al. (2017) | PCV3/KU-1609 |
| MF155642 | 2016 | China:Guangxi | PCV3 | Wen et al. (2017) | PCV3-China/GX2016-2 |
| MF155643 | 2016 | China:Guangxi | PCV3 | Wen et al. (2017) | PCV3-China/GX2016-3 |
| MF155641 | 2016 | China:Guangxi | PCV3 | Wen et al. (2017) | PCV3-China/GX2016-1 |
